# The mechanism of submandibular gland dysfunction after menopause may be associated with the ferroptosis

**DOI:** 10.18632/aging.103882

**Published:** 2020-11-05

**Authors:** Hyun-Keun Kwon, Ji Min Kim, Sung-Chan Shin, Eui-Suk Sung, Hyung-Sik Kim, Gi Cheol Park, Yong-Il Cheon, Jin-Choon Lee, Byung-Joo Lee

**Affiliations:** 1 Department of Otorhinolaryngology-Head and Neck Surgery, College of Medicine, Pusan National University and Medical Research Institute, Pusan National University Hospital, Busan, Korea; 2 Pusan National University Medical Research Institute, Pusan National University School of Medicine, Busan, Korea; 3 Department of Otorhinolaryngology-Head and Neck Surgery, College of Research Institute for Convergence of Biomedical Science and Technology, Pusan National University Yangsan Hospital, Yangsan, Korea; 4 Department of Life Science in Dentistry, School of Dentistry, Pusan National University, Yangsan, Korea; 5 Department of Otolaryngology-Head and Neck Surgery, Samsung Changwon Hospital, Sungkyunkwan University School of Medicine, Changwon, Korea

**Keywords:** ferroptosis, salivary gland, menopause, xerostomia

## Abstract

Salivary gland dysfunction is a common symptom that occurs after menopause. This study was performed to investigate the mechanism of salivary gland dysfunction to confirm the relationship between ferroptosis and salivary gland dysfunction by ovariectomy. Forty-eight female rats were randomly divided into four groups (12 rats in each group). Histology, real time PCR, western blot, immunohistochemistry, electron microscopy, cytosolic iron assay, and salivary function were analyzed. Human salivary gland tissue analysis was also done. Lipogenesis and lipid deposition in the submandibular gland tissue occurred after ovariectomy. ROS generation, MDA+HAE was increased and GPX4 activity was decreased and in the OVX group compared to the CON group. Iron deposition in the submandibular gland tissue was increased in the OVX group. Submandibular gland fibrosis was increased and saliva secretion was decreased in the OVX group. In human submandibular gland analysis, lipid and iron deposition was also increased in the postmenopause group. This is the first *in vivo* study in which salivary gland dysfunction is associated with the ferroptosis in postmenopausal animal model. Increased lipid and iron deposition in normal submandibular gland tissues of postmenopausal women can suggest that the salivary gland dysfunction after menopause may be associated with the ferroptosis.

## INTRODUCTION

Women over 50 years old inevitably experience menopause. Menopause is a physiological process occurring in the fifth decade of life in women, and involving permanent cessation of menstruation [1]. Menopause causes various changes such as hot flashes, osteoporosis, depression, cognitive impairment, voice change and weight gain [2–5]. Changes in the oral cavity also occur. Sex hormone changes in menopause may affect significant decrease of salivary flow, resulting in hypo-salivation and xerostomia [6]. Xerostomia can cause difficulty in swallowing and speaking, dental caries, altered taste, halitosis and burning mouth [7, 8]. Therefore, xerostomia reduces the quality of life of women after menopause.

Xerostomia is defined as the subjective perception of dry mouth [9]. Xerostomia is more common in middle-age and elderly people and more common in women than in men [10, 11]. The etiology of xerostomia varies and can be divided into local factors and systemic factors. Local factors include medication, head and neck radiotherapy, and lifestyle including smoking and drinking. Systemic factors include endocrine, autoimmune, infectious, and granulomatous diseases, and aging and menopause are also included [12]. Although there have been studies of xerostomia caused by radiotherapy, little is known about the mechanism of xerostomia that occurs after menopause. Topical agents and systemic sialogogues are used to treat xerostomia, but there is no ideal treatment. This is because the mechanism of xerostomia is not known yet.

Ferroptosis is a form of regulated cell death that is dependent on iron and reactive oxygen species (ROS) and is characterized by lipid peroxidation. It is morphologically and biochemically distinct and disparate from other processes of cell death [13]. Ferroptosis causes neurodegenerative disorders and has been reported as a mechanism of brain damage after intracerebral hemorrhage [14, 15]. The level of serum iron increase markedly after menopause and estrogen deficiency in postmenopausal women increases the expression of genes involved in lipogenesis [16–18]. Serum iron overloading and lipogenesis can induce ROS production and lipid peroxidation which is a main feature of ferroptosis. Thus, the authors hypothesized that ferroptosis may be related to the mechanism of xerostomia that occurs after menopause. This study was performed to investigate the histological and molecular mechanism of salivary gland dysfunction in the ovariectomized animal model and to confirm the relationship between ferroptosis and salivary gland dysfunction by ovariectomy.

## RESULTS

### Serum sex-hormone concentration and expression of sex-hormone receptors

**Figure 1:**
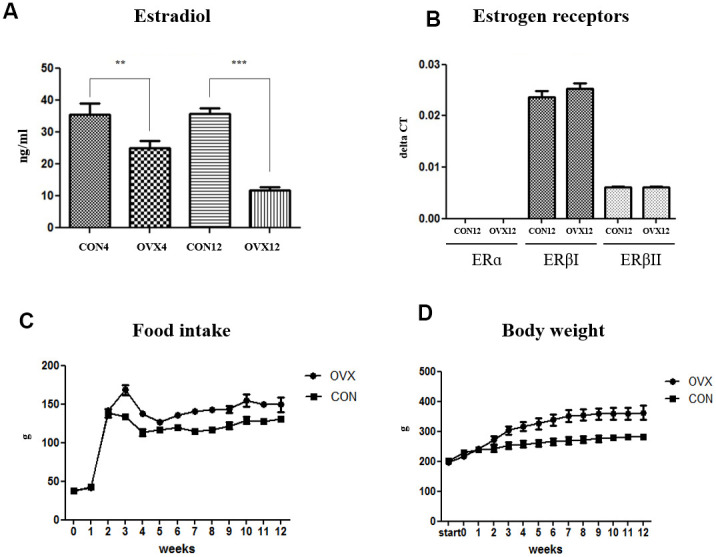
**Sex hormone and body weight change after ovariectomy.** (**A**) The level of serum estradiol concentration in the CON and OVX groups. (**B**) The expression of estrogen receptors in the CON12 and OVX12 group. (**C**), (**D**) Food intake and body weight in the CON and OVX group. Two-way ANOVA test. *p<0.05, **p<0.01 and ***p<0.001. ER = estrogen receptor, CON = control, OVX = ovariectomy.

The level of serum E2 concentration significantly decreased in the OVX4 group (p<0.01) and the OVX12 group (p<0.001) compared to the CON group (Figure 1A). Expression of estrogen receptors were not significantly different in the OVX group compared to the CON group. To evaluate the effect of sex hormone on sex hormone receptor, we performed real time PCR for the expression of estrogen receptors (ERs) such as ERα, ERβI, and ERβII in submandibular gland. ERα did not exist on submandibular gland. Although the ERβI and ERβII were expressed in submandibular gland, the expression of these receptors showed no significant differences in the OVX groups compared with the CON groups (Figure 1B). Compared with CON group, food intake was increased in OVX group and body weight was increased as a result (Figure 1C, 1D).

### Lipid deposition in submandibular gland

**Figure 2:**
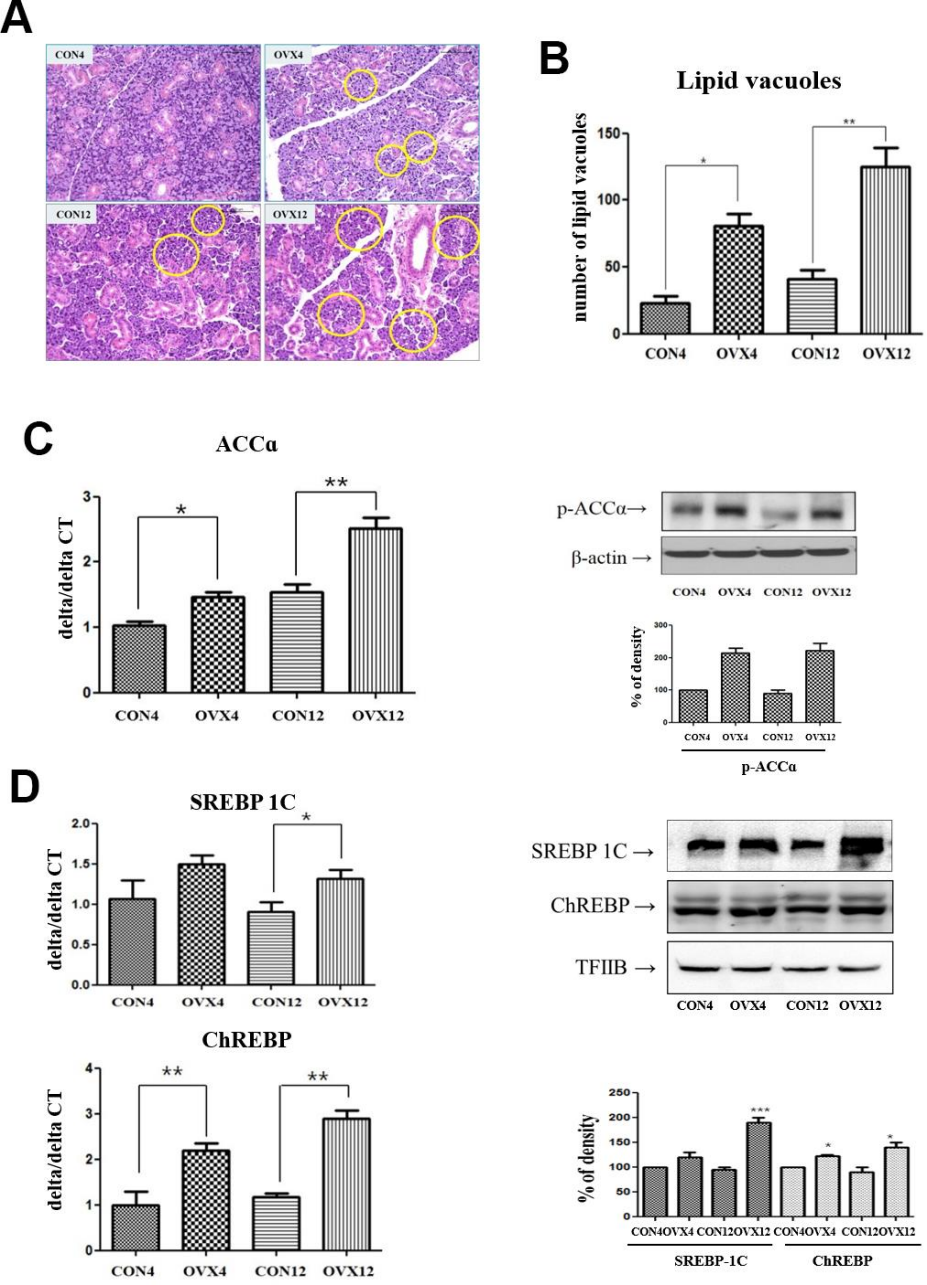
**Lipid deposition in submandibular gland and lipogenesis analysis.** (**A**) Lipid vacuoles (yellow circle) of submandibular gland detected by H & E staining. (**B**) Morphometric analysis of lipid vacuoles in the CON and OVX groups. (**C**), (**D**) Real time PCR and western blots of lipogenesis –related gene; ACC alpha and transcription factors; SREBP-1C and ChREBP expression in the CON and OVX groups. Two-way ANOVA test. *p<0.05, **p<0.01 and ***p<0.001. CON = control, OVX = ovariectomy, ACC = Acetyl-CoA carboxylase, SREBP = sterol regulatory element-binding protein, ChREBP = carbohydrate response element-binding protein.

To measure the deposition of lipid in submandibular gland, we examined with H&E stain (40X). In morphometric analysis, the number of lipid vacuoles of submandibular gland in the OVX4 group (80.16±9.56) and the OVX12 group (122.65±17.64) was significantly higher compared with the CON4 group (24.32±6.88) and the CON12 group (40.44±11.36) (p<0.05 and p<0.01) (Figure 2A, 2B). This result means lipid deposition in the submandibular gland tissue occurred by menopause.

### Lipid metabolism

We investigated the expression of Acetyl-CoA carboxylase (ACC), β-actin, carbohydrate response element-binding protein (ChREBP), and sterol regulatory element-binding protein (SREBP) for lipogenesis by rear time PCR and western blot. ACC was significantly increased in the OVX4 group (p<0.05) and the OVX 12 group (p<0.01) compared to the CON group. In western blot, p-ACCα expression was increased in the OVX group compared to the CON group. However, β-actin expression was not significantly different between two groups (Figure 2C). ChREBP was significantly increased in the OVX4 group (p<0.01) and the OVX12 group (p<0.01) compared to the CON group (Figure 2D). SREBP-1 was significantly increased in the OVX12 group (p<0.05) compared to the CON group. In western blot, SREBP-1C and ChREBP were increased in the OVX group compared to the CON group (Figure 2D).

### Electron microscopy

**Figure 3:**
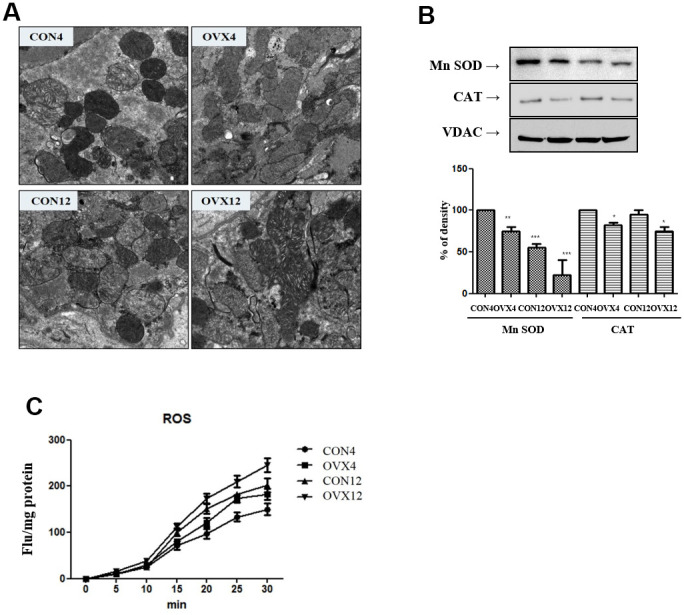
**Mitochondria dysfunction.** (**A**) Electron microscopy of submandibular gland in the CON and OVX groups. (**B**) Western blotting of antioxidant enzyme; SOD and catalase and control VDAC in the CON and OVX groups. (**C**) ROS generation in the CON and OVX groups. CON = control, OVX = ovariectomy, SOD = superoxide dismutase, CAT = catalase, VDAC = voltage-dependent anion channel.

Electron microscopy shows shrunken mitochondria, decreased cristae and ruptured outer membrane. Unlike CON4 and CON12 group, OVX4 and OVX12 groups show swollen mitochondria and a progressive loss of cristae. In the OVX groups, a disintegrated dead cell is presented (Figure 3A).

### Generation of ROS

In western blot, superoxide dismutase (Mn-SOD) and catalase, which can detoxify excess ROS, were decreased in the OVX group compared to the CON group (Figure 3B). As a result, it was confirmed that ROS generation was significantly increased in the OVX4 group (p<0.05) and the OVX12 group (p<0.01) compared to the CON group (Figure 3C).

### Redox imbalance and lipid peroxidation products

**Figure 4:**
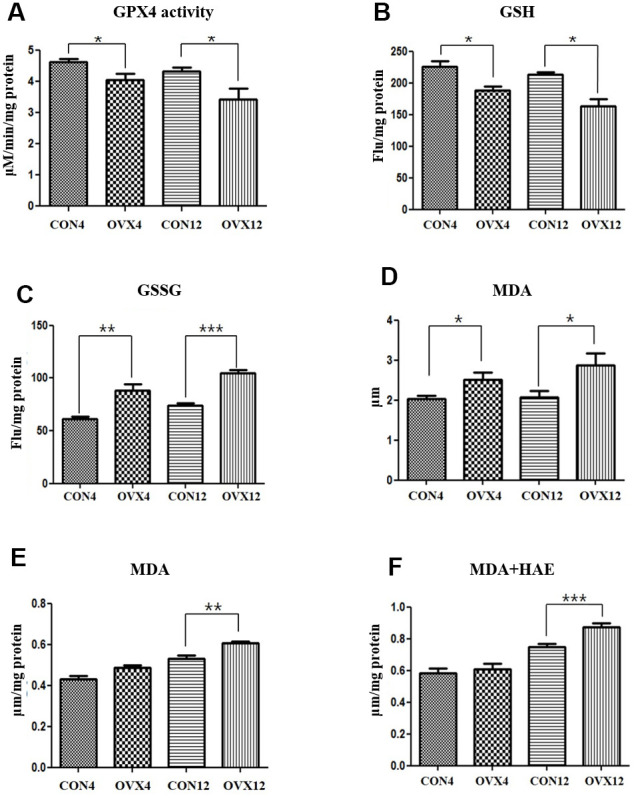
**Lipid peroxidation.** (**A–C**) GPX activity (**A**), GSH level (**B**) and GSSH level (**C**) in the CON and OVX groups. (**D**) MDA concentration in the serum in the CON and OVX groups. (**E**) MDA concentration in the submandibular gland tissue in the CON and OVX groups. (**F**) MDA and HAE concentration in the submandibular gland tissue in the CON and OVX groups. Two-way ANOVA test. *p<0.05, **p<0.01 and ***p<0.001. CON = control, OVX = ovariectomy, GPX = Glutathione peroxidase, GSH = glutathione, GSSG = oxidized GSH, MDA = malondialdehyde, HAE = hydroxyalkenals

GPX4 is an antioxidant enzyme that protects cells against membrane lipid peroxidation. GSH is a cofactor of GPX4 to catalyze the reduction of lipid peroxides. GPX4 activity and GSH level was significantly decreased in the OVX4 group (p<0.05) and the OVX12 group (p<0.05) compared to the CON group (Figure 4A, 4B). GSSH level was significantly increased in the OVX4 group (p<0.01) and the OVX12 group (p<0.001) compared to the CON group (Figure 4C). To confirm the increase of lipid peroxidation, the concentrations of MDA and 4-HNE, the lipid peroxidation products, were measured. MDA concentration in the serum was significantly increased in the OVX4 group (p<0.05) and the OVX12 group (p<0.05) compared to the CON group (Figure 4D). MDA and 4-HAE concentration in the salivary gland tissue was significantly increased in the OVX12 group (p<0.001) compared to the CON12 group (Figure 4E, 4F).

### Iron overload in submandibular gland

**Figure 5:**
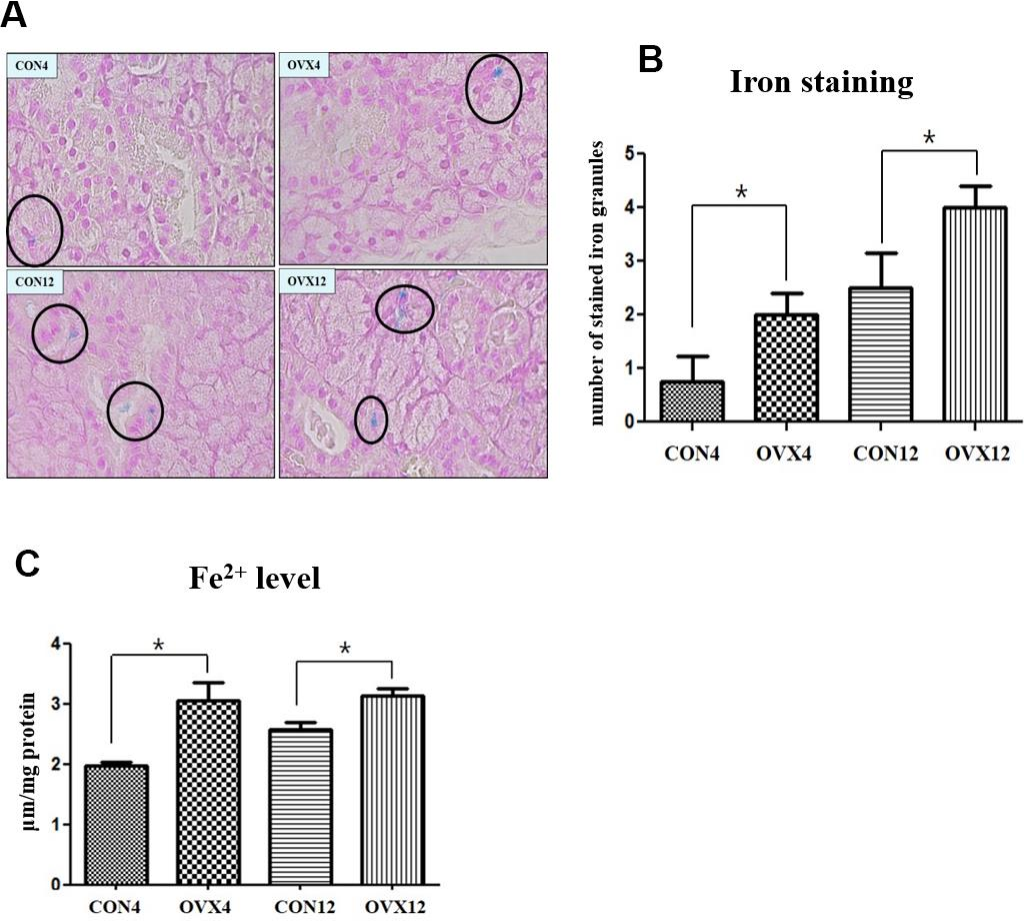
**Iron deposition in submandibular gland.** (**A**) The number of stained iron (black circle) detected by Prussian Blue iron staining. (**B**) Morphometric analysis of stained iron in the CON and OVX groups. (**C**) Cytosolic iron content in the CON and OVX groups. Two-way ANOVA test. *p<0.05, **p<0.01 and ***p<0.001. CON = control, OVX = ovariectomy.

To explore whether ovariectomy is associated with iron overload, we measured stained iron in tissue and cytosolic iron concentrations on submandibular gland. The number of stained iron of submandibular gland tissue in the OVX4 group (2.00±0.82) and the OVX12 group (4.00±0.82) was significantly higher compared with the CON4 group (0.75±0.96) and the CON12 group (2.50±1.29) (p<0.05) (Figure 5A, 5B). The cytosolic iron levels of the OVX groups were upregulated compared to the CON groups (p<0.05) (Figure 5C). These results indicate that the submandibular gland is overloaded with iron after ovariectomy.

### Inflammation cytokines secretion and fibrosis

**Figure 6:**
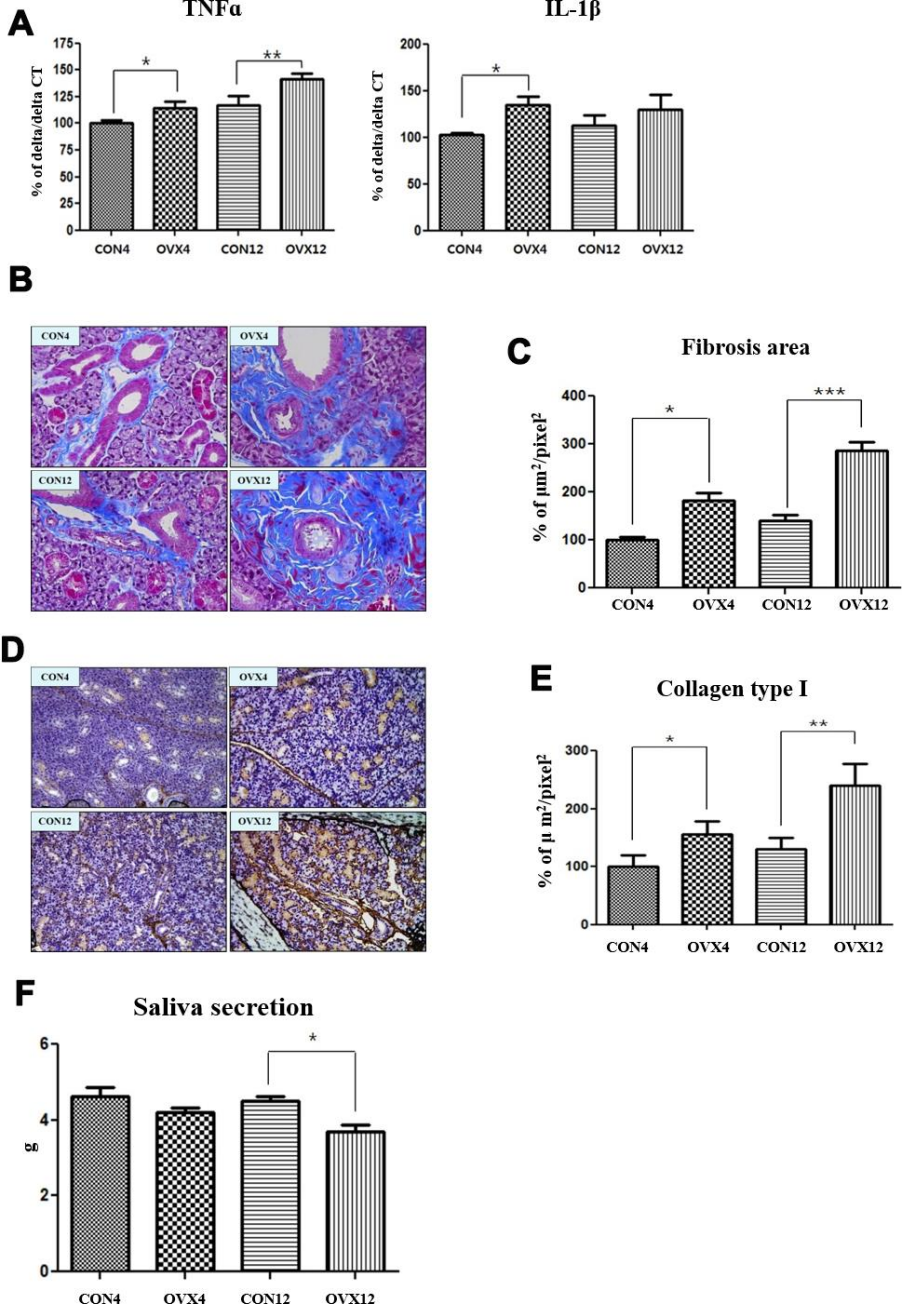
**Increased salivary gland fibrosis and decreased salivary gland function.** (**A**) Real time PCR of TNFα and IL-1β mRNA expression in the CON and OVX groups. (**B**) Fibrosis of submandibular gland detected by Masson and trichrome's staining. (**C**) Morphometric analysis of fibrosis in submandibular gland tissue in the CON and OVX groups. (**D**) Collagen type I expression of submandibular gland detected by immunohistochemistry. (**E**) Morphometric analysis of expression of collagen type I in the CON and OVX groups. (**F**) Saliva secretion in the CON and OVX groups. Two-way ANOVA test. *p<0.05, **p<0.01 and ***p<0.001. CON = control, OVX = ovariectomy, TNF = tumor necrosis factor, IL = interleukin.

Excess ROS causes oxidative stress which is associated with inflammation. The secretion of tumor necrosis factor alpha (TNF-α) was significantly increased in the OVX4 group (p<0.05) and the OVX12 group (p<0.01) compared to the CON group (Figure 6A). The secretion of interleukin 1 beta (IL-1β) was significantly increased in the OVX4 group (p<0.05) compared to the CON4 group (Figure 6A).

Morphometric analysis was performed to confirm fibrosis of submandibular gland tissue. In MT stain, fibrosis in submandibular gland tissue was significantly increased in the OVX4 group (p<0.05) and the OVX12 group (p<0.001) compared to the CON group (Figure 6B, 6C). In IHC, the expression of collagen type I was significantly increased in the OVX4 group (p<0.05) and the OVX12 group (p<0.01) compared to the CON group (Figure 6D, 6E).

### Saliva secretion

Saliva secretion was decreased in the OVX4 group and OVX12 group compared to the CON group. However, saliva secretion was significantly decreased only in the OVX12 group (p<0.05) (Figure 6F).

### Microscopic findings of human submandibular gland tissue

**Figure 7:**
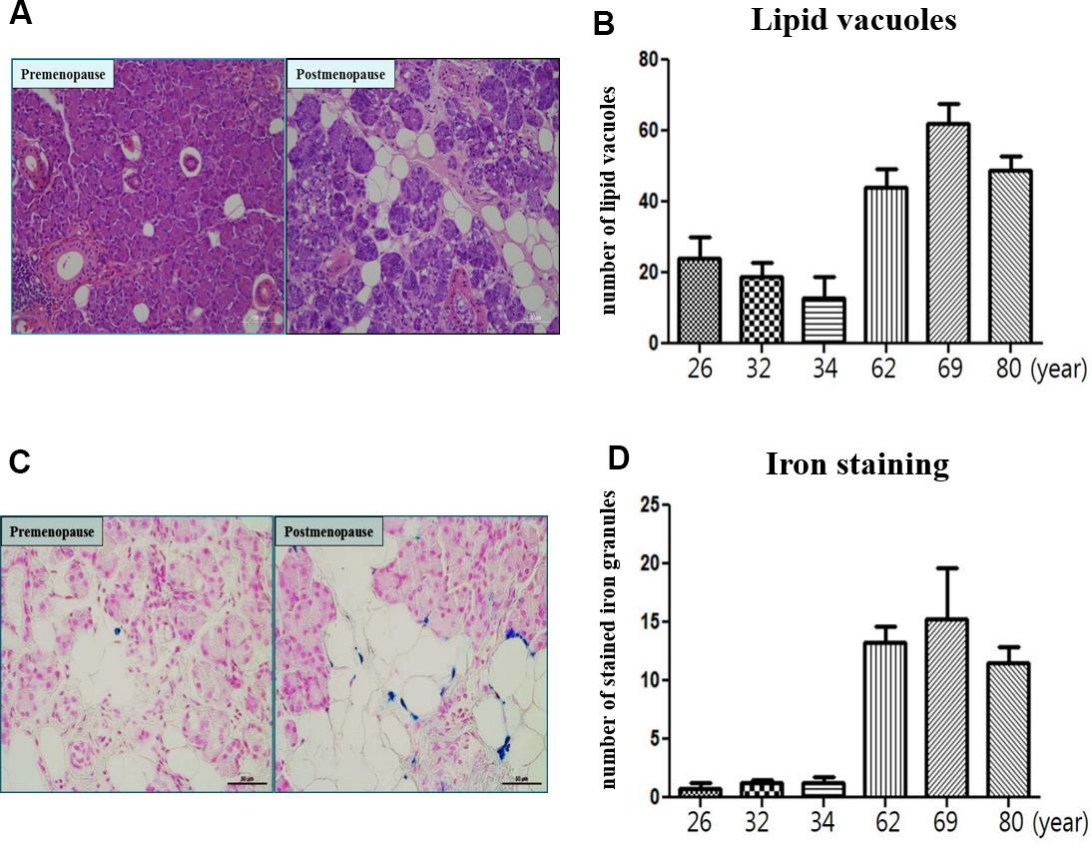
**Microscopic finding of human submandibular gland tissue.** (**A**) Lipid vacuoles of submandibular gland detected by H & E staining in the postmenopause group than premenopause group. (**B**) The number of lipid vacuoles of submandibular gland in the postmenopause group than premenopause group. (**C**) Iron accumulation of submandibular gland detected by Prussian Blue iron staining in the postmenopause group than premenopause group. (**D**) The number of stained iron of submandibular gland in the postmenopause group than premenopause group.

In morphometric analysis, the number of lipid vacuoles of submandibular gland in the postmenopause group (62year; 44.16±6.65, 69year; 63.36±7.02 and 80year; 48.15±3.85) was significantly higher compared to the premenopause group (26year; 22.15±7.12, 32year; 18.72±4.65 and 34year; 11.84±9.33) (Figure 7A, 7B). The number of stained iron of submandibular gland in the postmenopause group (62year; 13.25±2.00, 69year; 16.25±5.00 and 80year; 11.25±2.00) was significantly higher compared to the premenopause group (26year; 1.15±0.50, 32year; 1.55±0.25 and 34year; 1.50±0.02) (Figure 7C, 7D).

## DISCUSSION

Post-menopausal survival is increasing in women with increasing life expectancy. Dry mouth, which can occur after menopause, is one of the important symptoms that affect the quality of life in postmenopausal women. There is no effective standard treatment for xerostomia. Several mechanisms such as atrophy, oxidative stress, or apoptosis of salivary gland have been reported as a mechanism of dysfunction of salivary gland after menopause [19–22]. However, there is still no clear study of the mechanism of salivary gland dysfunction after menopause. Identifying the pathogenesis of salivary gland dysfunction caused by menopause is very important in developing novel therapeutic methods for xerostomia.

Although there have been some reports about increased lipid accumulation in the salivary glands by menopause, no studies have yet been conducted on the mechanisms and effects of lipid accumulation in salivary gland [23]. In this study, the reduction of estrogen by ovariectomy in experimental animal caused obesity by the increase of food intake. Weight gain is similar to changes in the women body that usually occur after menopause. The expression of key transcriptional regulators, such as SREBP-1 and CHREBP involved in lipid and glucose metabolism was increased by increasing obesity and food intake after ovariectomy. Increased expression of these transcriptional factors increases ACC alpha, important enzymes for lipogenesis. As a result, lipid accumulation increased in salivary gland after menopause.

The salivary gland steatosis, which accumulates fat in the salivary glands due to menopause, may be similar to the mechanism of cellular damage caused by fatty liver. After menopause, similar to fatty liver or nonalcoholic fatty liver disease, salivary gland becomes a fatty salivary gland or fatty salivary gland dysfunction. There are many studies on the mechanism of cell damage by lipids accumulation in hepatocytes [24–27]. Lipid accumulation impairs the oxidative capacity of mitochondria, produces excessive ROS, and causes morphological changes of mitochondria [27]. Excessive ROS production is also associated with the decrease of antioxidant factors of mitochondria. In this study, mitochondria were morphologically changed, such as swollen with loss of cristea. In addition, the reduction of various antioxidant enzymes, such as, superoxide dismutase (Mn-SOD), GSH, and catalase, and excessive ROS generation were observed. These findings are thought to be mitochondria dysfunction due to fatty salivary gland similar to fatty liver.

The lipid hydroperoxidase GPX4 converts lipid hydroperoxidase to lipid alcohols, and this process prevents the iron-dependent formation of toxic lipid ROS. Decreased GPX4 activity leads to increased lipid ROS formation and lipid peroxidation [28]. The dysfunction of mitochondria, the decrease of GPX4 due to the decrease of GSH, and the production of excessive ROS increased lipid peroxidation [29, 30]. In this study, MDA was increased by lipid peroxidation. Lipid peroxidation increases the secretion of various inflammatory cytokines and eventually leads to tissue fibrosis in the fatty liver [31, 32]. In this study, lipid peroxidation was increased in postmenopausal salivary glands, resulting in increased inflammatory cytokines such as IL-1 and TNF-α in salivary gland tissues, and fibrosis also increased. This finding suggests that postmenopausal salivary gland dysfunction is associated with lipid peroxidation by lipid accumulation and increased inflammatory cytokines with fibrosis.

**Figure 8:**
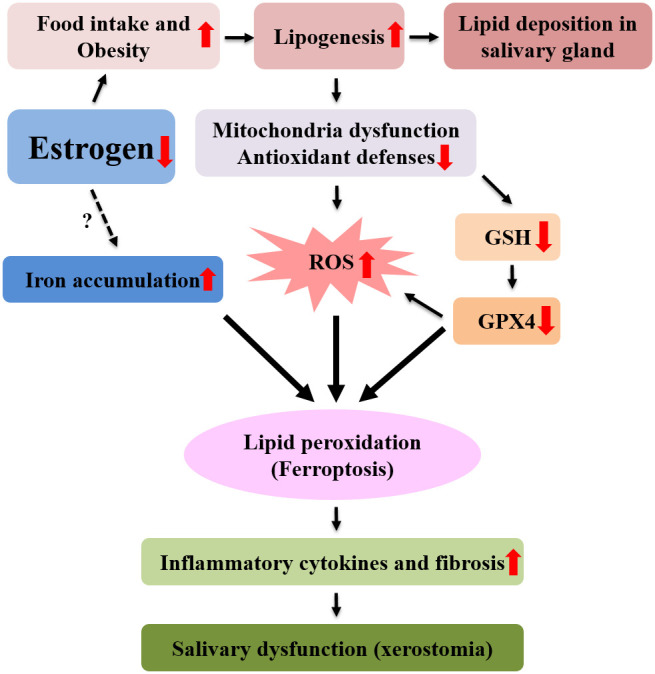
**Mechanism of postmenopausal xerostomia.** After menopause, lipid ROS production increased due to increased lipogenesis and lipid peroxidation, resulting in ferroptosis and fibrosis and inflammation of submandibular gland. As a result, the function of the submandibular gland is reduced, resulting in xerostomia.

Some studies show that iron increases in bone or heart tissue after menopause [16, 33, 34]. In this study, the tissue of post-menopausal salivary gland also increased iron. Increased lipid peroxidation and iron accumulation in postmenopausal salivary gland tissues indicate that postmenopausal xerostomia is a salivary gland dysfunction by ferroptosis. Ferroptosis was used for the first time to describe cell death characterized by iron-dependent accumulation of lipid ROS [35]. Ferroptotic death is morphologically, biochemically, and genetically distinct from apoptotic and nonapoptotic death due to the central involvement of iron-dependent lipid ROS accumulation [28, 35]. The features of ferroptosis are as follows : (a) the generation of ROS, (b) the depletion of GPX4 in cells (c) the accumulation of lipid hydroperoxides and the (d) the availability of iron [13]. All these findings were observed in the salivary gland after ovariectomy. After ovariectomy in experimental animal, food intake increased and obesity occurred due to the decrease of estrogen. Lipid accumulation, dysfunction of mitochondria, increased ROS production, decreased antioxidant factors including GSH, decreased GPX4, increased lipid peroxidation, and accumulation of iron were observed in salivary gland tissue after ovariectomy. These findings are consistent with ferroptosis. After ovariectomy, the ferroptosis of salivary gland increases the secretion of inflammatory cytokines, causing fibrosis in tissues, and finally results in salivary gland dysfunction and xerostomia (Figure 8).

In postmenopausal women, lipid and iron deposition in normal salivary gland obtained during submandibular gland resection are increased compared with young patients. Normal salivary gland tissue obtained after submandibular gland resection for benign submandibular gland tumor in postmenopausal women is thought to have effects on aging as well as menopause. There are no specific markers that can characterize the ferroptosis in paraffin-fixed salivary tissue slide. Increased lipid and iron deposition in normal submandibular gland tissues of postmenopausal women are not direct evidence of the ferroptosis, but it is speculated that the salivary gland dysfunction caused by menopause in women may be associated with the ferroptosis.

The pathogenesis of the various symptoms that occur in the reduction of estrogen after menopause is not clear. This study was the first to report the possibility of the ferroptosis as a mechanism of salivary gland dysfunction after menopause. Ferroptosis, a newly introduced cell death mechanism, is being studied as novel therapeutic target in several cancers cell lines and as one of pathogenesis in neurodegenerative disease, tuberculosis and heart disease [14, 36–40]. Physical changes caused by menopause occur slowly over a long period of time. Salivary gland dysfunction that occurs after menopause is thought to be due to the ferroptosis slowly occurred for three months in animal models of rats. The change of salivary gland in the 3 months after ovariectomy is a good chronic animal model of the ferroptosis.

This study is an *in vivo* study in which salivary gland dysfunction is associated with the ferroptosis in postmenopausal animal rats. *In vitro* studies of the exact mechanism of diminished function of the salivary gland by the reduction of estrogen during the process of the ferroptosis are necessary. However, established *in vitro* studies of cellular changes following estrogen reduction in normal salivary gland cell lines have not been reported. After menopause, the lack of suitable *in vitro* study design to mimic the change of salivary gland function after menopause is regarded as a limitation of this study. After menopause, further experimental or clinical studies on the effects of salivary glands function by various antioxidants, iron chelating agent, or inhibitors of ferroptosis are needed.

## MATERIALS AND METHODS

### Animals

Forty-eight female Sprague-Dawley rats (Samtako, Osan, Korea) aged nine weeks were used in this study. Animal care and research protocols were based on the principles and guidelines adopted by Guide for the Care and Use of Laboratory Animals (National Institutes of Health publication).

### Experimental design

After a week acclimatization period, rats were randomly divided into four groups (12 rats in each group): Group I (4 weeks after sham operation rats as CON4), group II (4 weeks after ovariectomy rats as OVX4), group III (12 weeks after sham operation rats as CON12) and group IV (12 weeks after ovariectomy rats as OVX12). Each animal group was weight matched at the beginning of the study. Rats were sacrificed four weeks (CON4 and OVX4) and twelve weeks (CON12 and OVX12) after surgery.

### Establishment of the ovariectomized rat model

The rats were anesthetized using isoflurane inhalation (3% dissolved in oxygen). Sham operation and ovariectomy (OVX) was performed as follows: rats were anesthetized, abdominal incision was made at the midline of the abdomen and bilateral ovaries being revealed. In the OVX groups, the ovaries were ligated and cut off bilaterally followed by closure of the abdominal cavity. In the sham operations groups, the ovaries were exposed and their anatomical position was examined, then the abdomen was closed without excision of ovaries.

### Plasma sex hormones analysis

Concentration of estradiol (E2) in serum was measured by rat-specific estradiol Enzyme-linked immunosorbent assay plates coated with biotin-conjugated binding protein kit purchased from Calbiotech Incorporation (Spring Valley, CA, USA).

### Histology and morphometric analysis

The submandibular glands were isolated from each rat and prepared for fixation overnight in 4% formalin. We used automatic tissue processor for paraffin embedding (Leica, TP1020, semi-enclosed benchtop tissue processor) and dispensing (Leica EG1150H, Heated paraffin embedding module). Cross-sections (8μm thick) were placed on slide glasses, and sections were prepared for Hematoxylin and Eosin (H&E), Masson and trichrome’s (MT) and Iron staining. Morphometric determination was undertaken at 40X pictures from whole part of submandibular gland tissues by menu of the morphometric method using image analysis system program composed of a light microscope (Leica Basic LAS V3.8 software).

### Prussian blue iron staining

The method based on the Prussian Blue stain reaction in which ionic iron reacts with acid ferrocyanide producing a blue color. Prussian blue reaction involves the treatment of sections with acid solutions of ferrocyanides. Any ferric ion present in the tissue combines with the ferrocyanide and results in the formation of a bright blue pigment called Prussian blue, or ferric ferrocyanide. Deparaffinize and rehydrate sections incubate for 3 min in iron stain (mix of potassium ferrocyanide and hydrochloric acid) and rinse in water. Counterstain in nuclear fast red and dehydrate in alcohol and mount.

### Tissue preparations

Frozen submandibular gland tissue were homogenized in hypotonic lysis buffer using a tissue homogenizer for 20 sec. Homogenates were kept on ice for 15 min, 125 μl of 10% Nonidet P-40 (NP-40) solution was added and mixed for 15 second, and the mixture was centrifuged at 14,000 *g* for 2 min. The supernatants were used as the cytosol fraction. The pelleted nuclei were washed once with 400 μl of buffer A plus 25 μl of 10% NP-40, centrifuged, suspended in 200 μl of nuclear buffer, kept on ice for 30 min, and centrifuged at 14,000 *g* for 10 min. The supernatant (nuclear protein) was harvested and then stored at -80°C (Kim *et al.* 2010). Protein concentration was measured by the bicinchonic Acid (BCA) assay.

### Real time-qPCR

To confirm mRNA expression, we isolated whole submandibular gland tissue and used real time PCR method. Tissue RNA was extracted using the TRIzol system (Life Technologies, Rockville, MD). Reverse Transcription Kit (Applied Biosystems, Foster City. California) was used to perform reverse transcription according to the manufacturer's protocol. Real-time PCR was performed according to the SYBR Green PCR protocol (Applied Biosystems Foster City CA). Gene-specific PCR products were continuously measured by an ABI PRISM 7900 HT Sequence Detection System (PE Applied Biosystem Norwalk, CT). All the primers used for Real Time PCR analysis have been designated using Primer Express software 1.5 (Applied Biosystems, Foster City, CA), and synthesized by Invitrogen Life Technologies (Carlsbad, CA). Primer sequences can be found in Table 1.

**Table 1:** Sequence of primers.

ERα (estrogen receptor α)	Forward	GCCTTCTACAGGTCCAATTCTGAC
	Reverse	ACAGCACAGTAGCGAGTCTCC
ERβI (estrogen receptor βI)	Forward	GCTTCGTGGAGCTCAGCCTG
	Reverse	AGGATCATGGCCTTGACACAGA
ERβII (estrogen receptor βII)	Forward	GAAGCTGAACCACCCAATGT
	Reverse	CAGTCCCACCATTAGCACCT
ACCα (acetyl-CoA carboxylase alpha)	Forward	GACGTTCGCCATAACCAAGT
	Reverse	CTGCAGGTTCTCAATGCAAA
ChREBP (Carbohydrate responsive element binding protein)	Forward	CAGGATGCAGTCCCTGAAAT
	Reverse	GAGGTGGCCTAGGTGGTGTA
SREBP 1C (Sterol regulatory element-binding protein 1)	Forward	GGCCCTGTGTGTACTGGTCT
	Reverse	AGCATCAGAGGGAGTGAGGA
*18s RNA*	Forward	AACCCGTTGAACCCCATT
	Reverse	GGGCAGGGACTTAATCAACG

### Western blot

Western blotting was carried out as described previously. The membrane was incubated with specific primary antibody at 25°C for 3 hr, followed by a horseradish peroxidase-conjugated anti-mouse antibody (Santa Cruz, 1:10,000), an anti-rabbit antibody (Santa Cruz, 1:10,000), or an anti-goat antibody (Santa Cruz, 1:10,000) at 25°C for 1 hr. Antibody labeling was detected using West-zol Plus and chemiluminescence Fluorchem^TM^SP (Alpha Innotech Corporation, San Leandro, CA, USA).

### Quantitation of redox status and lipid peroxidation

ROS generation was measured with specific fluorescence probe. Briefly, 25 μM of 2′, 7′-DCF-DA was added to homogenates to a 250 μl final volume. Changes in fluorescence intensity were measured every 5 min for 30 min on a fluorescence plate reader, GENios (Tecan Instruments, Salzburg, Austria) with excitation and emission wavelengths set at 485 and 530 nm, respectively. For the assay to measure glutathione (GSH) levels, 1 mM EDTA-50 mM phosphate buffer was added to the supernatant of trichloric acid (TCA)-treated homogenates, followed by *o*-phthaldehyde, and the mixture was incubated for 25 min at room temperature. To assay oxidized GSH (GSSG) level, N-ethylmaleimide was added to the supernatant of TCA-treated homogenates, then incubated for 25 min. Both GSH and GSSG levels were measured at excitation and emission wavelengths set at 360 and 460 nm, respectively. Glutathione peroxidase 4 (GPX4) activity was investigated with a Glutathione Peroxidase Assay Kit (Abcam, USA). Malondialdehyde (MDA)/4-hydroxyalkenals (HAE) concentrations were determined through use of a Bioxytech LPO-586 Assay Kit (OXIS Health Products, Foster, CA, USA). The kit uses a chromatogenic reagent that reacts with the lipid peroxidation products, MDA and 4-HAE, yielding a stable chromophore with maximum absorbance at 586 nm.

### Cytosolic iron assay

Concentration of iron in serum was measured by rat-specific colorimetric iron assay kit from Biovision Incorporation (Spring Valley, CA, USA).

### Immunohistochemistry (IHC)

De-paraffinized sections were washed with PBS and blocked for 1 hour at room temperature with 2% BSA containing 0.3% Triton X-100 in PBS. They were then incubated for 24 hours at 4°C with the following primary anti-collagen I (Abcam, Cambridge, United Kingdom). After the primary antibody was removed by rinsing, sections were incubated with secondary antibodies for 1 hour at room temperature. The following goat-anti rabbit secondary antibodies were used for double-staining with DAB staining. Incubation with phosphate-buffered saline supplemented with 1% bovine serum albumin instead of the primary antibody served as a negative control. For immunohistochemical analysis of submandibular gland, three nonoverlapping areas were analyzed at 400X, and nine areas were analyzed in each section. Results were expressed as stained area per total area in micrometers squared. Data were expressed as medians and ranges.

### Electron microscopy

The material was pre-fixed with 2.5% glutaraldehyde (4°C, phosphate buffer, pH 7.4) and was post-fixed with 1% osmuim tetroxide in the same buffer. The material dehydrated with a series of the graded ethyl alcohol, and embedded in epoxy resin (Epon 812 mixture). Thick sections (1 *μ*m) were stained with 1% toluidine blue for light microscope. Thin sections (50~60 nm) were prepared by using an ultramicrotome (EM UC7, Leica) and were double stained with uranyl acetate and lead citrate. Thin sections were examined with a transmission electron microscope (JEM-1200EXII, JEOL). The morphological feature of mitochondria was observed using electron microscopy.

### Saliva secretion

Saliva secretion was induced by subcutaneous injection of pilocarpine (2 mg/kg body weight). The saliva was collected using cotton balls between 5 to 30 min after pilocarpine injection under deep anesthesia with isoflurane inhalation (3% dissolved in oxygen). The total weight of the secreted saliva (difference in the weight of the cotton balls before and after collection) was measured.

### Human tissue analysis

In the submandibular gland of the patient who underwent submandibular resection due to submandibular gland disease, a tissue slide of the normal part was analyzed. We observed lipid vacuole and iron deposition in the submandibular tissues of 3 premenopausal women (26, 32 and 34 years old) and 3 postmenopausal women (62, 69 and 80 years old). This study was approved by the Institutional Review Board of Pusan National University Hospital (IRB No. H-1910-031-084) and informed consent was waived.

### Statistical analysis

Unless otherwise noted, all quantitative data were reported as the mean standard error of the mean from at least three parallel repeats. Two-way analysis of variance (ANOVA) was used to determine significant differences between groups in which P < 0.05 was considered statistically significant. For Western blotting, one representative blot is shown from independent experiments done in triplicate.
